# Whole-Genome Sequencing of SARS-CoV-2 from Quarantine Hotel Outbreak

**DOI:** 10.3201/eid2708.204875

**Published:** 2021-08

**Authors:** Lex E.X. Leong, Julien Soubrier, Mark Turra, Emma Denehy, Luke Walters, Karin Kassahn, Geoff Higgins, Tom Dodd, Robert Hall, Katina D’Onise, Nicola Spurrier, Ivan Bastian, Chuan K. Lim

**Affiliations:** University of South Australia, Adelaide, South Australia, Australia (L.E.X. Leong);; South Australian Health and Medical Research Institute, Adelaide (L.E.X. Leong);; SA Pathology, Adelaide (L.E.X. Leong, J. Soubrier, M. Turra, L. Walters, K. Kassahn, G. Higgins, T. Dodd, I. Bastian, C.K. Lim);; University of Adelaide, Adelaide (J. Soubrier, G. Higgins, I. Bastian, C.K. Lim);; Department for Health and Wellbeing, Adelaide (E. Denehy, R. Hall, K. D’Onise, N. Spurrier);; Royal Adelaide Hospital, Adelaide (C.K. Lim)

**Keywords:** SARS-CoV-2, COVID-19, severe acute respiratory syndrome coronavirus 2, viruses, respiratory infections, zoonoses, vaccines, Australia, coronavirus disease, hotel quarantine, genome sequencing

## Abstract

Hotel quarantine for international travelers has been used to prevent coronavirus disease spread into Australia. A quarantine hotel–associated community outbreak was detected in South Australia. Real-time genomic sequencing enabled rapid confirmation tracking the outbreak to a recently returned traveler and linked 2 cases of infection in travelers at the same facility.

Since November 2019, community outbreaks of coronavirus disease (COVID-19), seeded by uncontrolled local transmission of severe acute respiratory syndrome coronavirus 2 (SARS-CoV-2) after importations, have overwhelmed the healthcare systems in many countries (*1*). Australia, including the state of South Australia, has largely controlled local transmission through early public health control measures such as rapid contact tracing and extensive nucleic acid amplification testing (NAAT). To limit introduction of SARS-CoV-2 into Australia, state and territory governments mandated 14-day quarantine in dedicated facilities for returning international travelers, including SARS-CoV-2 testing on arrival and before release. The clinically supervised hotel system enables containment of the risk for transmission associated with these travelers, especially those coming from countries experiencing SARS-CoV-2 resurgence (*2*) and asymptomatic or presymptomatic viral shedding carriers (*3*).

No local transmission had been recorded in the state since August 2020 until a community outbreak was identified in early November; the outbreak numbered 33 epidemiologically clustered cases as of December 2020. The first identified positive case in the outbreak was a family member of a housekeeping attendant at one of the quarantine hotels. Immediate screening of close contacts rapidly identified 14 additional cases, including 2 security guards working in the same hotel. The suspected source case was a traveler returned from the United Kingdom. Two additional cases were in travelers who stayed in rooms adjacent to that source patient. We hypothesized that this outbreak might have been caused by an inadequate ventilation system of the quarantine hotel.

Our study detailed the laboratory aspect of the quarantine hotel–associated outbreak, highlighting the utility of genomic sequencing for detecting the source of infection in locally acquired cases. Before this outbreak, all SARS-CoV-2–positive isolates in South Australia, including those from internationally returned travelers, had been prospectively sequenced on the Illumina platform (https://www/illumina.com) using the tiled amplicon ARTIC primers (https://github.com/artic-network/artic-ncov2019/tree/master/primer_schemes/nCoV-2019/V1) directly on clinical specimens. Sequencing reads were then aligned to the reference genome (Wuhan-Hu-1, RefSeq NC_045512.2) for construction of consensus genomes. A comprehensive database of high-quality SARS-CoV-2 genomes, representing >81% of positive isolates in South Australia, was thus available for comparison. Most (28/33) consensus genomes from this outbreak comprised >1,000× read depth and >95% coverage of the reference genome.

We designated the quarantine hotel–associated outbreak variant as B.1.36.1 lineage using the pangolin nomenclature system (*4*) (pangolin version 2.3.8, pangoLEARN v2021-04-01; https://github.com/cov-lineages/pangolin). The phylogenetic tree with genomes from its parental lineage B.1.36 (n = 3,010) suggested that this lineage and its sublineages potentially emerged from its ancestral lineage in February 2020 (Figure). To date, the B.1.36 lineage in GISAID has a large representation of genome sequences from the United Kingdom (n = 1,864, 62%), South Asia (n = 445, 15%), Europe (n = 324, 11%) and the Middle East (n = 155, 5%) (*7*).

**Figure:**
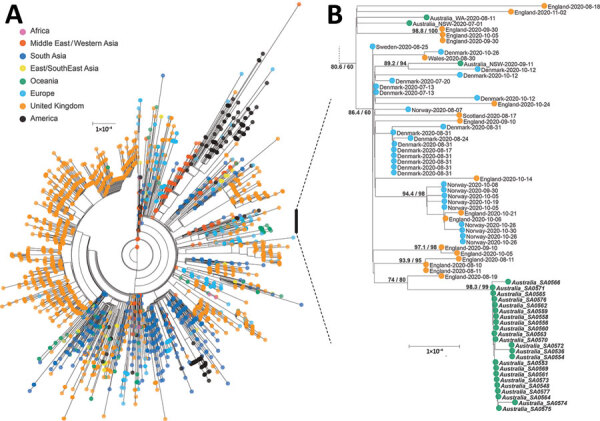
Maximum-likelihood phylogenetic tree of severe acute respiratory syndrome coronavirus 2 genomes from a quarantine hotel–associated community outbreak of coronavirus disease in South Australia, Australia. A) Genomes from lineage B.1.36 (n = 3,038). B) Subtree of lineage B.1.36.1 focusing on the quarantine hotel clusters and a returned traveler from the United Kingdom; bold type indicates those strains. Consensus genomes were profile-aligned using COVID-Align (*5*), and phylogenetic trees were constructed using IQ-TREE with general time reversible plus invariate plus gamma 4 sites model (*6*). SH-like approximate likelihood ratio test score was 98.3%, ultrafast bootstrap approximation 99%. Scale bar indicates substitutions per site.

Phylogenetic analysis using the 3,038 consensus genomes from the B.1.36 lineage, including those from this outbreak, demonstrated that the outbreak cluster represented 1 highly supported distinct clade consisting of all genomes from the quarantine hotel–associated cases (Figure). Although the source case was asymptomatic at the time of arrival, their mandatory nasopharyngeal swab showed relatively high viral loads by quantitative PCR (E-gene cycle threshold = 27.52); antinucleocapsid antibody seroconversion 14 days later confirmed the timing of infection.

Transmission within the initial large family group with a high infection rate demonstrated the overdispersion characteristics of SARS-CoV-2 (*8*). The short viral incubation period and generation time for this clonal SARS-CoV-2 cluster reduced our capacity to predict the transmission chain within this outbreak. Another limitation for a thorough epidemiologic investigation of the source case is the extended distance between the South Australia cluster and other UK cases on the phylogenetic tree, which is likely due to gaps in global sequencing effort.

Mutational profile analysis showed limited evidence in this outbreak variant to support enhanced infection or transmission from a single site mutation (*9*). Apart from the most notable D614G mutation for enhanced replication, this cluster does not have the mutations at the receptor binding motif of the spike protein that are common to several variants of concern (N417K/T, L452R, E484K/Q, N501Y/T).

Our study demonstrated that, in addition to hotel quarantine to prevent introduction of SARS-CoV-2 into the community, NAAT testing and rapid genomic sequencing are essential components of an effective public health response. In contrast to epidemiology-guided sequencing approaches, a comprehensive sequencing of all COVID-19 positive cases is important in discovering previously unidentified links. Sequencing enabled us to identify 2 additional case-patients who were guests in the quarantine hotel, which led to further improvements and policy changes in the quarantine system. This outbreak in the context of no recent local transmission highlights the transmissibility of SARS-CoV-2 and the risk for transmission chains that are occurring unchecked in countries with a high incidence of SARS-CoV-2.
